# Supplementary material for: Redox reactions and weak buffering capacity lead to acidification in the Chesapeake Bay
Source: Nat Commun. 2017 Aug 28;8:369. doi: 10.1038/s41467-017-00417-7 (PMC5573729; doi:10.1038/s41467-017-00417-7)
Supplement: Supplementary file 1 — Supplementary Information [file 41467_2017_417_MOESM1_ESM.pdf]

Description of Supplementary Files

Title: Supplementary Information

Description: Supplementary Figures and Supplementary References

Title: Peer Review File

Supplementary Figures

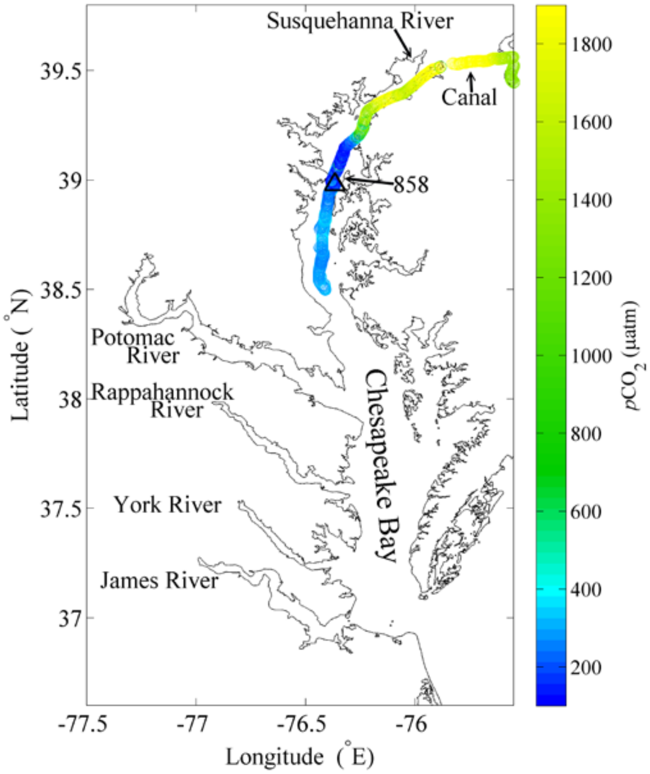

Supplementary Figure 1. Surface water *p*CO_2 (µatm) in the Chesapeake Bay during 18-24 August 2014. Data were collected underway aboard the R/V *Sharp* similar to Figure 1 in the main article. Station 858 is our focused study site. *p*CO_2 in the mid-bay (37.9 to 39.0°N) was undersaturated with respect to the atmosphere (~390 µatm) with an average value of 251.6±70.9 µatm in August 2014.

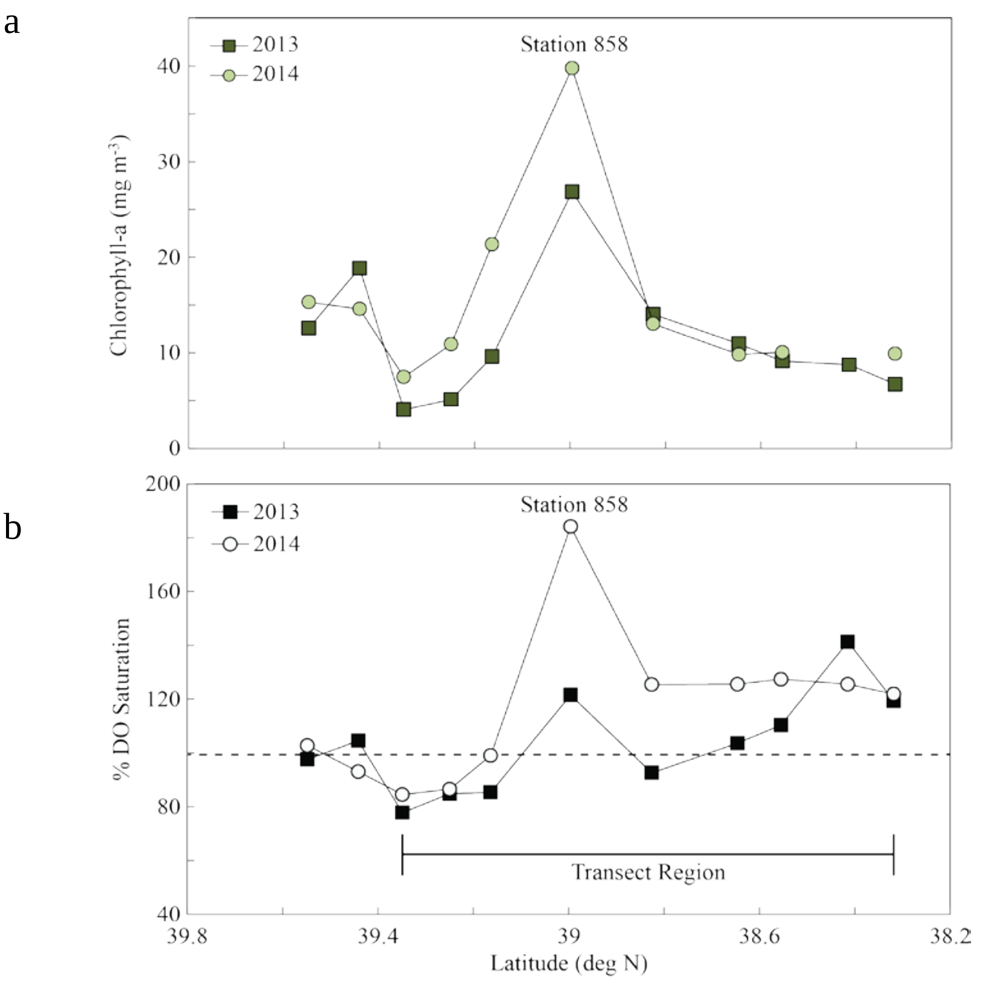

Supplementary Figure 2. Transects of chlorophyll-a and dissolved oxygen saturation (in %) measured in surface waters (0.5 m depth) along the axis of the main stem of the Chesapeake Bay between August 12-14, 2013 and August 4-6, 2014 (see http://www.chesapeakebay.net/data). The under-saturated oxygen levels north of 39.2 ^{o}N are consistent with super-saturated pCO_2 levels measured during the underway transect while the chlorophyll-a peak and over-saturated oxygen levels at 39 ^{o}N are consistent with extremely under-saturated pCO_2 levels.

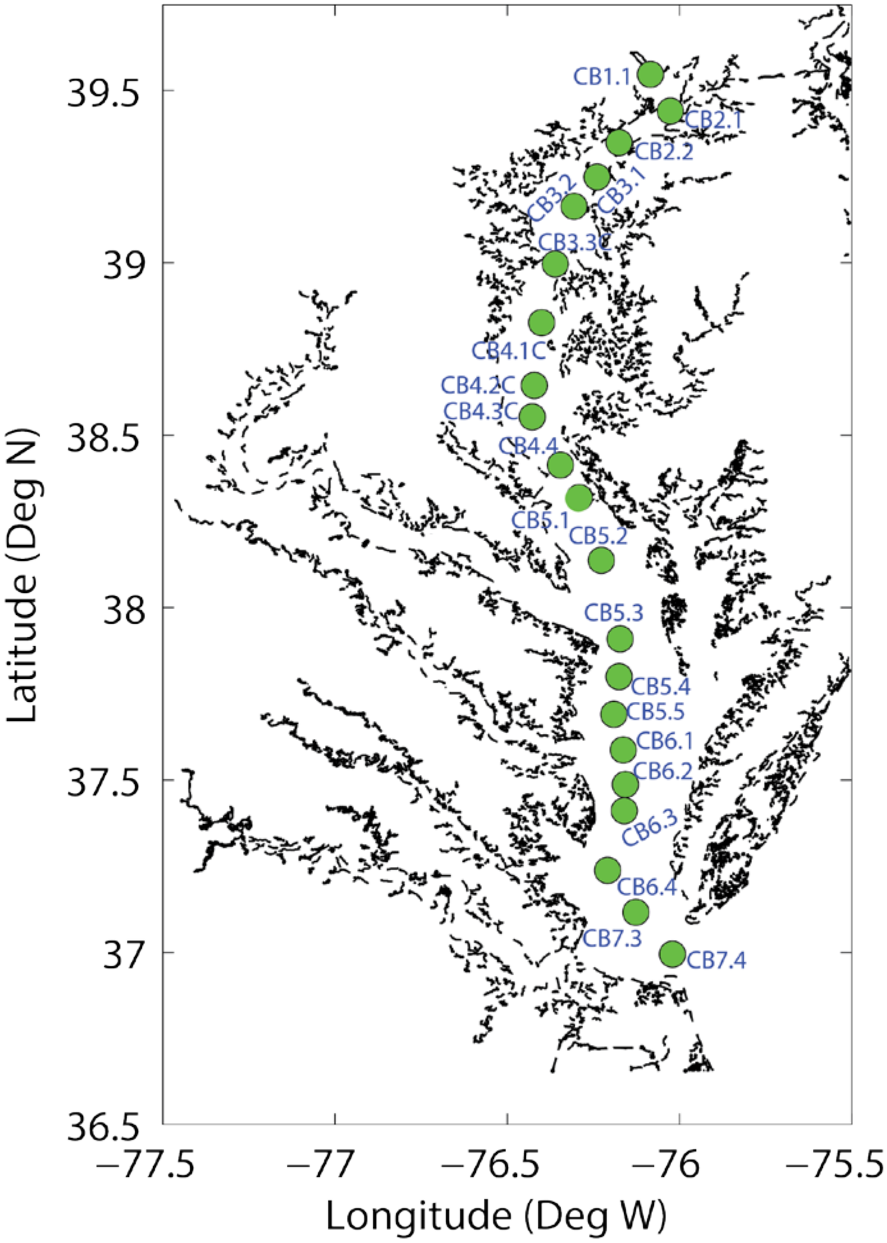

Supplementary Figure 3: Monitoring stations for measurements of surface (0.5 to 1 m) dissolved oxygen, temperature, and salinity in Chesapeake Bay, USA.

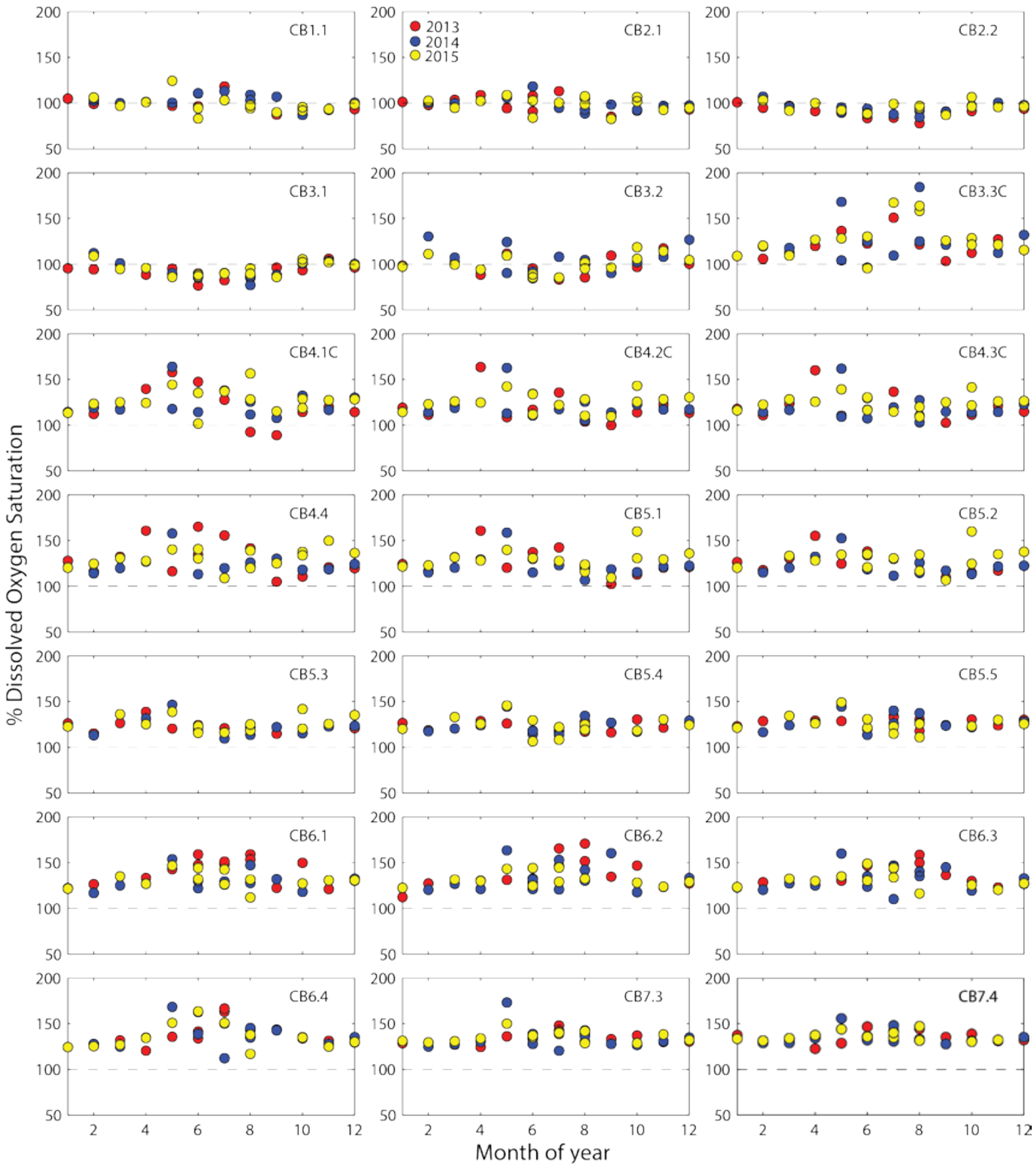

Supplementary Figure 4: Annual cycles of percent dissolved oxygen saturation at 21 stations in Chesapeake Bay (USA) for each year over the 2013-2015 period. Station locations are given in part 1. We analyzed monthly and regional patterns of percent dissolved oxygen saturation in surface waters of Chesapeake Bay for the years 2013, 2014, and 2015. The data originated from the long-term monitoring program in Chesapeake Bay, where we downloaded concentrations of dissolved oxygen and measurements of water temperature and salinity from the Chesapeake Bay Program website (www.chesapeakebay.net). We used the equations of Benson and Krause (1984)[1] to compute dissolved oxygen saturation for measurements made at bi-weekly to monthly intervals at 21 stations that span the longitudinal axis of the estuary. We averaged data for each month over the 2013-2015 period and plotted the along-axis pattern of percent saturation (this figure) as well as plotted the monthly averages for each year (2013, 2014, 2015) at all stations (Supplementary Figure 5). For further explanation is given in Methods.

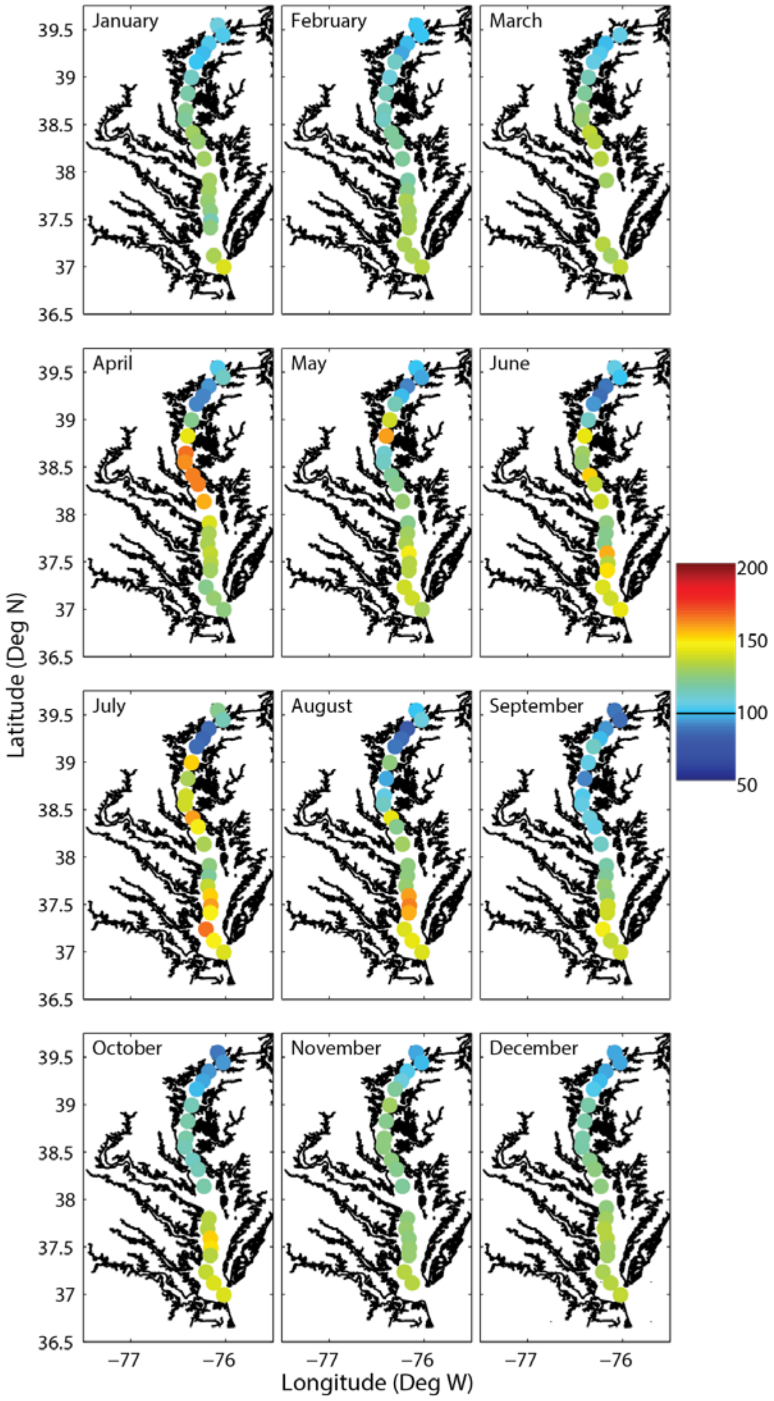

Supplementary Figure 5: Monthly percent dissolved oxygen saturation at 21 stations in Chesapeake Bay (USA) averaged over the 2013-2015 period. For further explanation, see Supplementary Figure 4 caption and Methods.

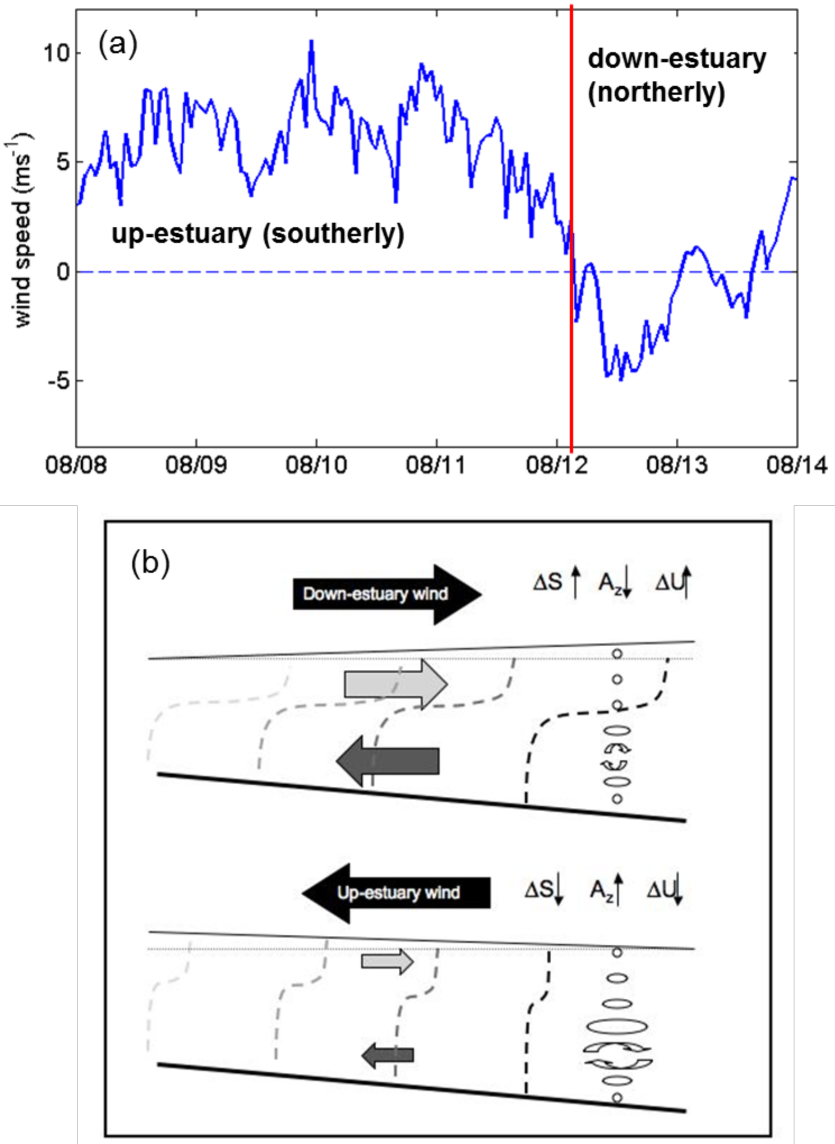

Supplementary Figure 6. A simplified picture of the physical conditions in Chesapeake Bay. (a) Time series of the along-channel wind speed during August 2013: the wind switched from southerly (up-estuary) to northerly (down-estuary). (b) A schematic diagram showing the straining effect of along-channel wind on estuarine stratification (ΔS): down-estuary wind enhances vertical shear (ΔU) and strains the along-channel density gradient to increase stratification and reduce vertical mixing (A_z), whereas up-estuary wind reduces vertical shear and stratification and increases vertical mixing. This is a reproduction of Fig. 6 in ref[2]. The strong southerly (up-estuary) wind during August 8-11 generated vertical mixing and tilted the isopycnals vertically to reduce stratification while the weak northerly (down-estuary) winds on August 12-13 strained the along-channel density gradient to increase stratification as shown in Fig. 2a in the main text. The data were collected at a mid-bay tower as part of a NSF-funded project[3].

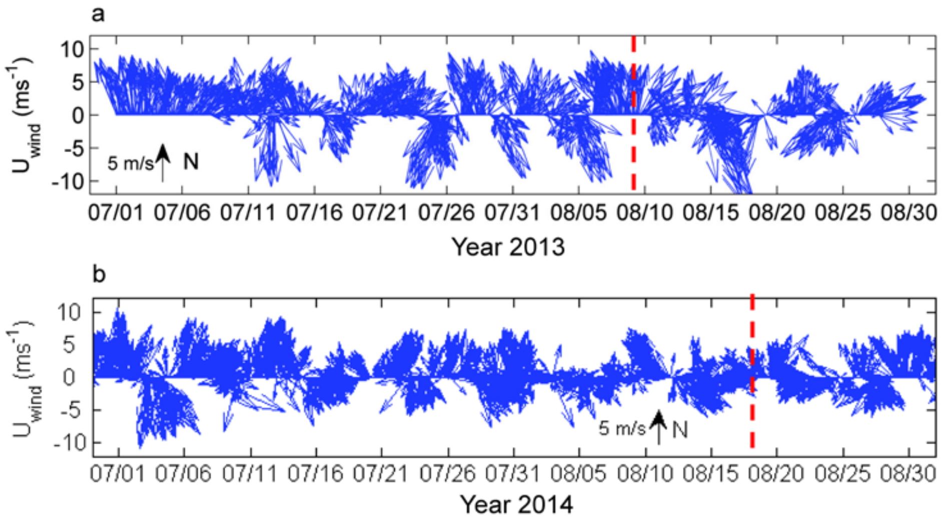

Supplementary Figure 7. Hourly wind speed vector at a mid-bay station before and during our cruises in (a) 2013 and (b) 2014. The cruise period was from August 9 to 14 in 2013 and August 18-24 in 2014. Red dashed line indicates day 1 of each cruise. It shows that wind direction shifts constantly between down-estuary (northerly, favoring re-stratification) and up-estuary (southerly, favoring mixing) directions during both years. However, overall wind speed was strong and changed direction before August 9, 2013 but was not as strong and as variable before August 18, 2014. The data were collected at a mid-bay tower[3].

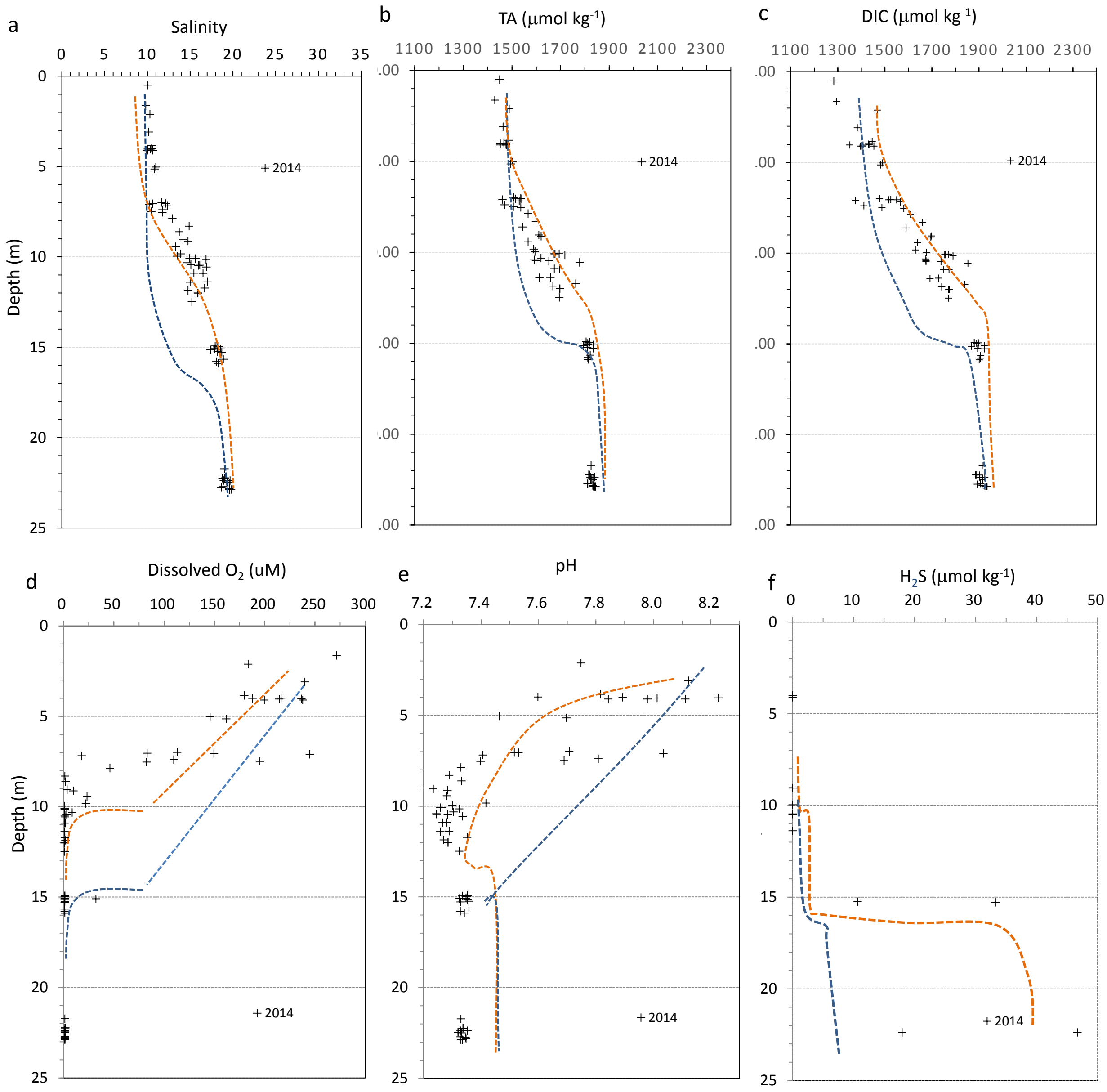

Supplementary Figure 8. Vertical distributions of measured chemical properties at the focused study site in 2014. (a) Salinity, (b) Total Alkalinity (TA), (c) Dissolved Inorganic Carbon (DIC), (d) Dissolved Oxygen, (e) pH (25°C and NBS scale), and (f) H_2S concentration. The lines are the lower and upper boundaries between days 1 & 2 and days 3-5, respectively from the 2013 cruise (that is Fig 2 in the main article) for a comparison.

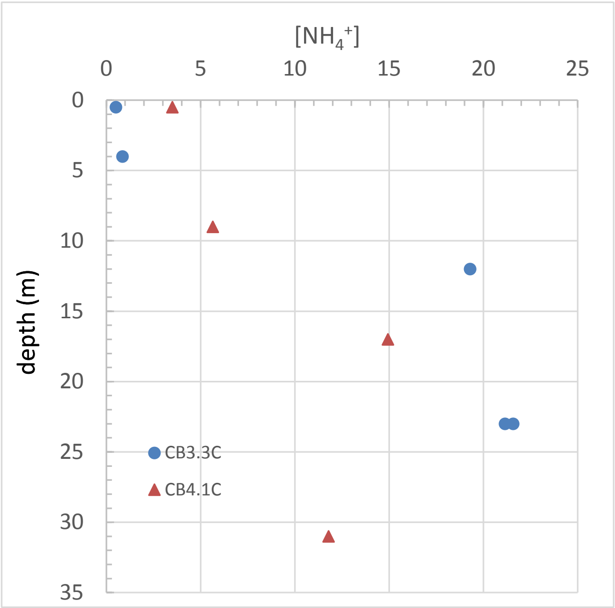

Supplementary Figure 9. Depth profiles of $[NH_4^+]$ at two mid-bay stations near our focused study site in August 13, 2013. Station locations (CB3.3C and CB4.1C) are given in Supplementary Figure 3. Data from the Chesapeake Bay Program; http://www.chesapeakebay.net/data/.

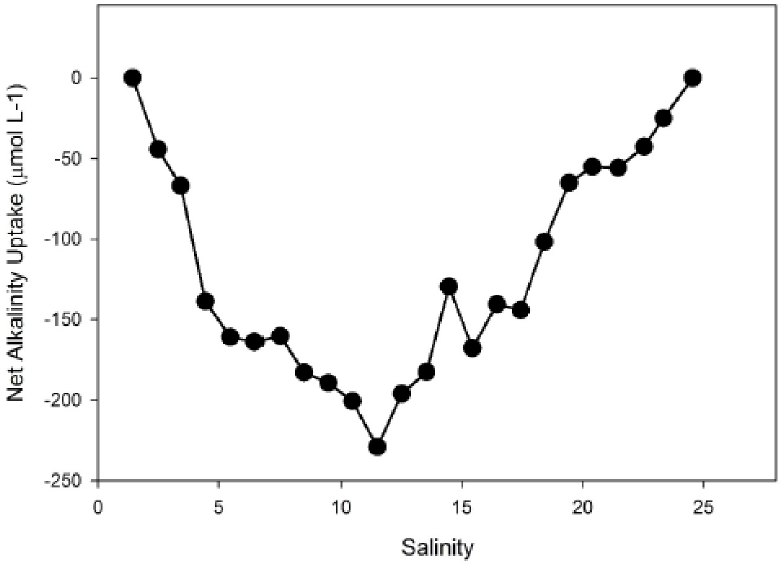

Supplementary Figure 10. Net alkalinity uptake in surface waters computed from data from 1985 to 2007 presented in Waldbusser et al. (2013)[4]. A conservative mixing line was generated between the lowest salinity samples and the highest salinity values in the Chesapeake Bay monitoring dataset. Deviations from that conservative mixing line indicate alkalinity uptake in surface waters, of a magnitude that matches with the estimated carbonate dissolution in deep water.

a b

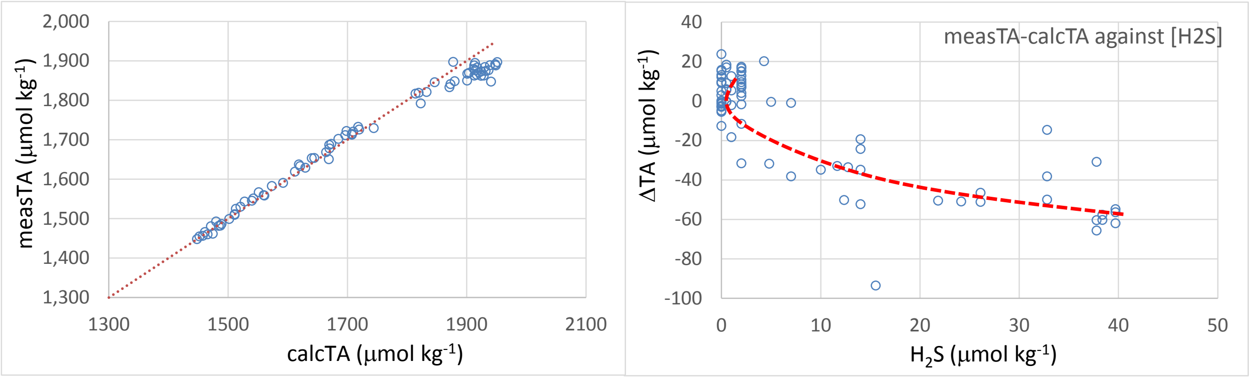

Supplementary Figure 11. Measured TA vs. calculated TA from DIC and pH. (a) A direct comparison. (b) The difference between measured and calculated vs [H_2S]. TA and DIC was measured after adding $HgCl_2$ for preservation while pH was measured on board without preservation.

Supplementary References

1 Benson, B. B. & Krause, D. The concentration and isotopic fractionation of oxygen dissolved in freshwater and seawater in equilibrium with the atmosphere. *Limnology and Oceanography* **29**, 620-632 (1984).

2 Scully, M. E., Friedrichs, C. & Brubaker, J. Control of estuarine stratification and mixing by wind-induced straining of the estuarine density field. *Estuaries* **28**, 321-326, doi:10.1007/bf02693915 (2005).

3 Fisher, A. W., Sanford, L. P. & Suttles, S. E. Wind Stress Dynamics in Chesapeake Bay: Spatiotemporal Variability and Wave Dependence in a Fetch-Limited Environment. *Journal of Physical Oceanography* **45**, 2679-2696, doi:doi:10.1175/JPO-D-15-0004.1 (2015).

4 Waldbusser, G. G., Powell, E. N. & Mann, R. Ecosystem effects of shell aggregations and cycling in coastal waters: an example of Chesapeake Bay oyster reefs. *Ecology* **94**, 895-903, doi:10.1890/12-1179.1 (2013).
